# Case Report: Observational use of mycophenolate mofetil in probable steroid-unresponsive immune checkpoint inhibitor-associated transverse myelitis

**DOI:** 10.3389/fimmu.2026.1933514

**Published:** 2026-09-21

**Authors:** Lingping Kong, Xin Wang, Tao Yu, Junping Wang, Chunsheng Yang, Linlin Zhang, Diansheng Zhong

**Affiliations:** 1 Department of Medical Oncology, Tianjin Medical University General Hospital, Tianjin, China; 2 Department of Imaging, Tianjin Medical University General Hospital, Tianjin, China; 3 Department of Neurology, Tianjin Medical University General Hospital, Tianjin, China

**Keywords:** immune checkpoint inhibitors, immune-related adverse events, pleural mesothelioma, steroid-unresponsive, transverse myelitis

## Abstract

Immune checkpoint inhibitor (ICI)-associated transverse myelitis (TM) is a rare and severe neurological immune-related adverse event (n-irAE) with an incidence of < 0.01%, and the clinical management of steroid-unresponsive cases remains extremely challenging. This article reports the case of a 37-year-old male patient with biphasic pleural mesothelioma who developed ICI-associated TM after treatment with nivolumab combined with ipilimumab. Initial treatment with high-dose glucocorticoids plus intravenous immunoglobulin (IVIg) was ineffective. After mycophenolate mofetil (MMF) was added in combination with steroids for 1 month, the patient’s neurological function showed slight improvement, and intramedullary contrast-enhanced signals on spinal MRI showed partial reduction. However, spinal cord cystic or myelomalacic changes suggested potentially irreversible parenchymal injury. Currently, the patient has restarted pemetrexed combined with carboplatin chemotherapy, and the disease status is stable. Based on recently published case series, case reports, and systematic reviews, this article discusses the second-line treatment strategies for steroid-unresponsive ICI-associated TM, aiming to provide a reference for clinical practice in managing such rare cases.

## Introduction

Immune checkpoint inhibitors (ICIs), including programmed death-1 (PD-1)/cytotoxic T lymphocyte-associated antigen 4 (CTLA-4) inhibitors, have become an important treatment for various malignant tumors, such as malignant pleural mesothelioma, significantly improving the survival of patients with advanced disease. However, while ICIs remove immune checkpoint-mediated inhibition of T cells and activate systemic antitumor immune responses, they inevitably induce immune-related adverse events (irAEs) involving multiple organ systems. Among them, neurological immune-related adverse events (n-irAEs) are relatively rare but can progress rapidly and are associated with high rates of disability, making them a key focus and challenge in clinical management (1). Transverse myelitis (TM) is one of the most severe types of ICI-associated n-irAEs, accounting for only 2% of all n-irAEs (2).

According to literature reports, the overall incidence of ICI-associated TM is < 0.01%, which occurs more commonly after treatment with PD-1/PD-L1 inhibitors alone or in combination with CTLA-4 inhibitors. The median time to onset is 6 weeks (range: 1–74 weeks), with 80% occurring within the first 4 months of treatment. Typical manifestations include limb weakness, abnormal sensory level, and sphincter dysfunction, which can result in permanent neurological deficits if not promptly treated (1, 2). To date, there have been relatively few reported cases of ICI-associated TM worldwide, reported mainly as case series or individual cases, among which approximately 30% are related to combined PD-1 inhibitor and CTLA-4 inhibitor therapy (3). Currently, the first-line treatment for ICI-associated TM is high-dose glucocorticoid pulse therapy, but some patients are steroid-refractory or steroid-dependent, requiring timely initiation of second-line immunosuppressive therapy. Existing second-line treatment options include intravenous immunoglobulin (IVIg) (4, 5), plasmapheresis, cyclophosphamide, rituximab, and other immunosuppressive therapies; however, no established standard exists (6, 7).

Mycophenolate mofetil (MMF), as a well-established immunosuppressant, has demonstrated efficacy in treating ICI-related hepatitis and nephritis (8). In addition, existing literature indicates that the pathogenesis of immune checkpoint inhibitor-associated TM is complex, involving abnormal T-cell activation, autoantibody production, and cytokine storms. For example, elevated interleukin-6 (IL-6) levels in the cerebrospinal fluid of some patients suggest a strong inflammatory response and cytokine-mediated tissue injury (9). In other cases, increased levels of chemokines such as C-X-C motif chemokine ligand 13 (CXCL13) in the cerebrospinal fluid indicate the involvement of B-cell-mediated immune responses. At the same time, some reported cases have a history of spinal radiotherapy, suggesting that radiotherapy may increase the risk of ICI-associated TM by damaging the blood-brain barrier and enhancing immune cell infiltration (7).

In the present case, the patient exhibited a suboptimal response to high-dose corticosteroid monotherapy. To address this therapeutic challenge, we added MMF as a second-line immunosuppressive agent. Mechanistically, MMF exerts its therapeutic effect by inhibiting inosine monophosphate dehydrogenase (IMPDH), thereby suppressing T and B lymphocyte proliferation, reducing cytokine production, and mitigating immune-mediated tissue injury within the spinal cord. By integrating the diagnostic and treatment course of this case with evidence from recently published case reports and reviews, this article analyzes the therapeutic strategies for steroid-unresponsive ICI-associated TM, providing a clinical basis and novel insights for the diagnosis and treatment of similar cases.

## Case

### General information and clinical background

A 37-year-old male patient with no previous medical history, no history of smoking or alcohol use, and no significant family history was admitted. In November 2022, the patient underwent a chest CT due to cough, which revealed small nodules in the upper lobe of the right lung and the dorsal segment of the lower lobe of the left lung (size approximately 9.6 mm × 6.2 mm × 5.6 mm), accompanied by calcification and interlobar pleural adhesion along the left oblique fissure. The nodules were considered benign calcified nodules. On 8 July 2025, the patient was admitted to the cardiology department of our hospital due to worsening chest pain. PET-CT showed an irregular soft tissue nodule in the subpleural area of the dorsal segment of the left lower lung, multiple enlarged lymph nodes in the left hilum and mediastinum (SUV = 11.0SUV), diffuse thickening of the left pleura (SUV = 7.9), and a large amount of left pleural effusion. On 23 July 2025, thoracoscopic pleural lesion resection was performed. The pathological diagnosis was a malignant tumor of the left parietal and visceral pleura, with biphasic mesothelioma (epithelioid-predominant) considered based on clinical and immunohistochemical findings.

### Tumor treatment and onset of irAE

#### ICI treatment phase

From 5 August to 21 October 2025, the patient received first-line treatment with nivolumab 360 mg d1 Q3W + ipilimumab 50 mg d1 Q6W, completing a total of four cycles. After three cycles, efficacy evaluation showed stable disease (SD), but left thoracoabdominal pain continued to worsen.

#### Pain treatment and chemotherapy

On 27 October 2025, intrathecal injection + spinal nerve root radiofrequency ablation was performed, but the analgesic effect was poor. On 14 November 2025, the patient received one cycle of pemetrexed combined with carboplatin chemotherapy at an outside institution.

#### Onset of neurological symptoms

On 20 November 2025, the patient suddenly developed bilateral lower limb weakness accompanied by sensory abnormalities and was unable to stand. On 24 November, the symptoms worsened, with the development of anterior waist girdle sensation. On 26 November, he was admitted to the neurology department.

### Clinical manifestations and specialist examination

On admission, specialist examination showed clear consciousness, normal articulation, and no abnormalities in cranial nerve examination. Limb muscle strength was Grade V in both upper limbs and Grade I in both lower limbs. Muscle tone was normal on the left side and decreased on the right side. There was abnormal superficial pain sensation in the left lower limb, decreased superficial pain sensation in the right lower limb, decreased sensation in the saddle area and posterior waist, and an obvious anterior waist girdle sensation. Deep sensation was decreased in both lower limbs. Bilateral hyperreflexia and a positive Babinski sign were present. Meningeal irritation signs were negative, and autonomous urination function was completely lost.

### Auxiliary examinations

#### Cerebrospinal fluid and serological examinations (27 November 2025)

Opening intracranial pressure was 122 mmH_2_O. Gross appearance of cerebrospinal fluid (CSF) was yellowish, with positive Pandy’s test (3+). Routine CSF showed a red blood cell count of 2 × 10^6^/L and a white blood cell count of 100 × 10^6^/L, with a lymphocyte predominance (mononuclear cells, 97%; multinuclear cells, 3%). CSF biochemistry showed a total protein level of 5.60 g/L (significantly elevated), chloride of 105 mmol/L (decreased), and glucose of 2.5 mmol/L (reference, 2.5–4.4 mmol/L). CSF oligoclonal bands were positive, while serum oligoclonal bands were negative. CSF cytology showed no malignant tumor cells. CSF-NGS detected 57 Epstein–Barr-virus-4 reads with a relative abundance of 99.1%; blood-NGS returned negative. No clinical or laboratory evidence supported overt acute central nervous system infection. Serological testing showed that anti-AQP4, anti-MOG, and anti-GFAP antibodies were negative in both serum and CSF. The paraneoplastic antibody panel showed serum anti-Ri immunoglobulin G (anti-Ri-IgG) positivity (1:32), and the remaining paraneoplastic antibodies were negative. No obvious abnormalities were observed in the autoantibody profile, thyroid function, or serum tumor markers.

#### Imaging examinations

##### Initial thoracic spinal MRI (27 November 2025)

Heterogeneous signals within the spinal canal at T2 to T10 levels, with linear prominent enhancement of the dura mater and arachnoid mater, irregular morphology of the spinal cord, and heterogeneous enhancement, consistent with TM inflammatory changes (**Figures 1A, C**).

**Figure 1:**
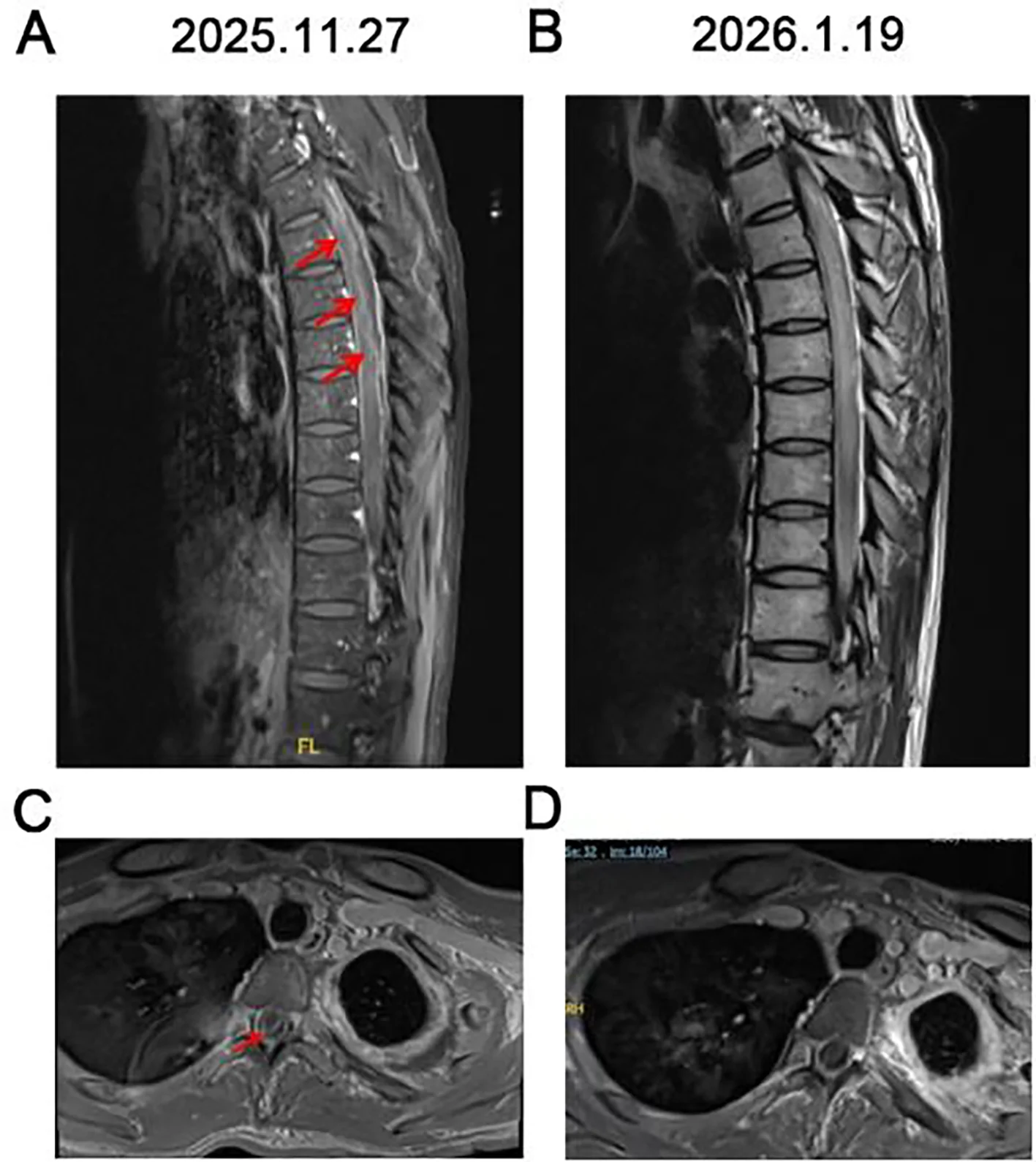
MRI progression. At diagnosis **(A, C)**, 1 month after combined steroid and MMF therapy **(B, D)**. **(A)** Sagittal contrast-enhanced T2-TSE sequence. Note that CSF demonstrates low signal intensity on this contrast-enhanced T2-TSE acquisition, with intramedullary inflammatory hyperintensity (red arrows). **(B)** Sagittal noncontrast T2-weighted TSE image showing partial reduction of intramedullary inflammatory signal abnormalities. **(C)** Axial postcontrast T1-weighted image demonstrating intramedullary inflammatory enhancement (red arrow). **(D)** Follow-up axial postcontrast T1-weighted image showing partial reduction of intramedullary enhancement.

### Diagnosis

Following a multidisciplinary consultation involving oncologists, neurologists, and radiologists, the radiologist pointed out that imaging studies after immune checkpoint inhibitor (ICI) therapy showed long-segment enhancement of the spinal meninges (dura mater and leptomeninges) and spinal cord, which made tumor metastasis less likely. Isolated Epstein-Barr virus (EBV) myelitis is rare and usually involves short segments when present, and imaging cannot reliably distinguish it from paraneoplastic lesions. The neurologist noted that cerebrospinal fluid examination showed markedly elevated protein, with glucose within the normal reference range. CSF cytology showed no malignant tumor cells, which makes malignant and infectious etiologies clinically less likely. Negative demyelinating antibodies ruled out autoimmune demyelinating diseases. The clinical presentation of long-segment myelitis accompanied by girdle-like pain and paralysis was consistent with typical immune-mediated injury. Although EBV infection and positive Ri antibody may act as triggering factors, the main pathogenic mechanism was ICI-induced immune dysfunction. Moreover, the treatment approach for TM is consistent across these three etiologies. The oncologist stated that, given the clear use of ICI therapy within 1 month before symptom onset and the atypical manifestation associated with Ri antibody positivity (which typically involves the brainstem and cerebellum), this case was diagnosed as ICI-associated TM, and a mixed multifactorial etiology could not be excluded. Multidisciplinary consultation favored ICI-associated TM as the predominant etiology, while mixed multifactorial contributions could not be fully ruled out.

### Treatment course and symptom changes

From 29 November to 3 December, the patient was treated with intravenous immunoglobulin (25 g daily for 5 days), which resulted in limited clinical response.

From 5 to 9 December, methylprednisolone was administered at 500 mg daily for 5 days, leading to partial relief of abdominal and subcutaneous pain. On 10 and 11 December, methylprednisolone 80 mg was given daily for 2 days, and the muscle strength of the right lower limb improved from Grade I to Grade II. On 11 December, methylprednisolone 40 mg was administered for 1 day, with no change in symptoms. On 12 December, the patient was switched to oral prednisone 30 mg daily, with a rapid tapering schedule that reduced the dose to two tablets every other day at the patient’s request. Symptoms remained unchanged during this period.

Subsequently, the patient was transferred to the Department of Medical Oncology for further treatment. On 15 December, serum cytokine levels were normal, while ferritin was elevated at 1,708.93 ng/ml. Sensory, motor, and autonomic nerve dysfunction persisted, indicating that there was no improvement in TM.

From 15 to 21 December, the patient received methylprednisolone 80 mg daily for 7 days combined with MMF 0.5 g twice daily. This resulted in partial recovery of the sensory deficit in the right lower limb.

From 22 to 29 December, oral prednisone 50 mg daily was administered for 7 days in combination with MMF 0.5 g twice daily. The sensory level on the right lower limb shifted caudally, and the muscle strength of the left lower limb improved from Grade I to Grade II.

During the follow-up period after discharge, prednisone was tapered by one tablet weekly, while MMF 0.5 g twice daily was continued. Symptoms did not improve further, and the abdominal band-like sensation persisted. Serum ferritin levels gradually decreased during treatment (**Figure 2**).

**Figure 2:**
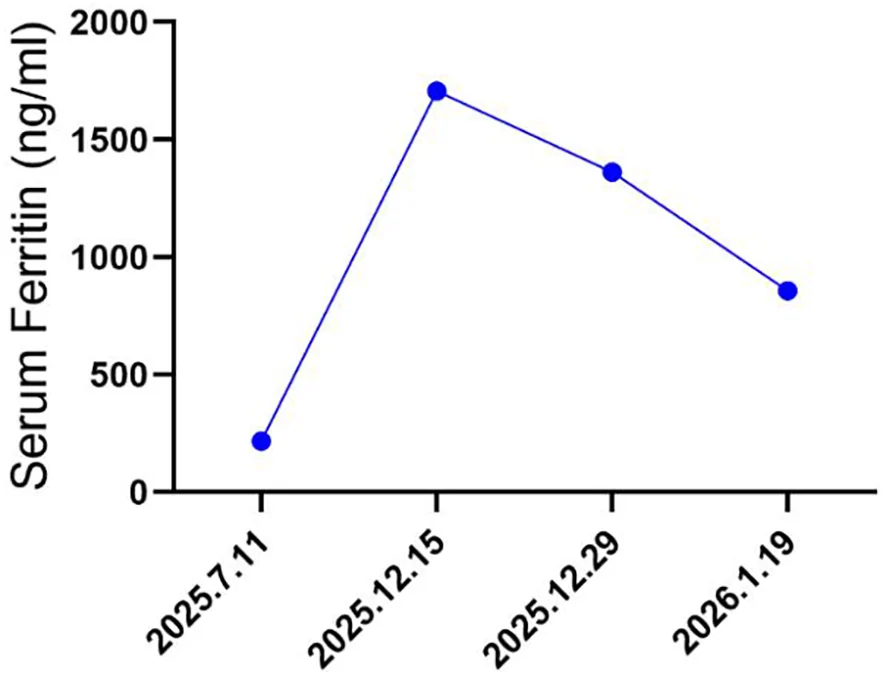
Trend of serum ferritin levels before and after treatment.

### Restart of tumor treatment

On 29 December, the patient received the second cycle of pemetrexed plus carboplatin chemotherapy. After evaluation, the patient’s neurological symptoms were stable, immunosuppressive treatment had not caused severe infection, spinal inflammatory lesions were controlled, and there was no evidence of tumor progression. Therefore, on the basis of continuing maintenance treatment with MMF + low-dose steroids, pemetrexed combined with carboplatin chemotherapy was restarted. During chemotherapy, neurological function, blood counts, liver and kidney function were closely monitored, and no severe chemotherapy-related adverse reactions occurred.

### Rechecked thoracic spinal MRI (19 January 2026, 1 month after treatment)

The intramedullary abnormal enhancement signals from T2 to T10 showed partial reduction in extent and intensity (**Figures 1B, D**); cystic or myelomalacic changes were observed within the previously affected spinal cord (**Figure 3**).

**Figure 3:**
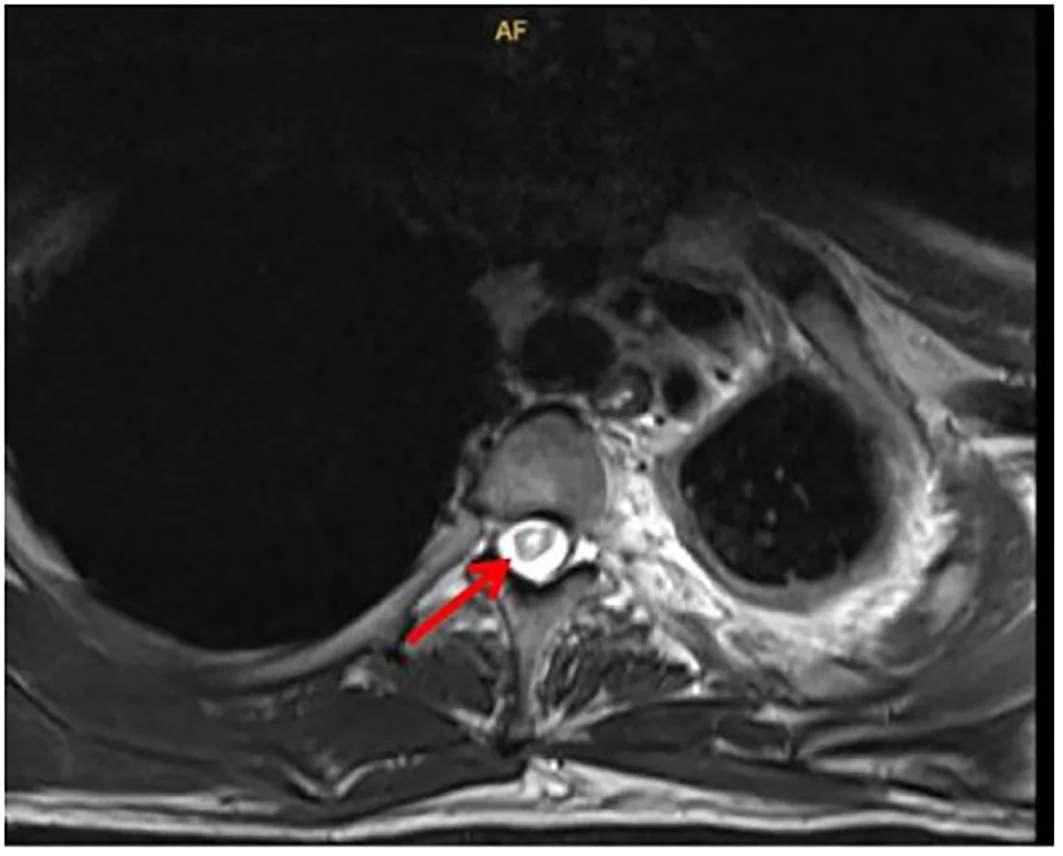
MRI demonstrated cystic or myelomalacic foci within the spinal cord.

### Chest CT (dynamic reexamination during treatment)

Nodular thickening of the left pleura and left pleural effusion showed no significant progression compared with the previous assessment; recent reexamination following the restart of chemotherapy (January 2026) showed stable tumor lesions with no new lesions.

### Outcome and prognosis

#### Neurological function outcome (1 month after treatment)

After 1 month of MMF combined with steroid treatment, the patient’s neurological function showed a partial recovery pattern: bilateral lower limb muscle strength recovered from Grade I to Grade II; superficial sensation in the right lower limb partially recovered, while the anterior waist girdle sensation did not improve; the area of decreased sensation in the saddle area and posterior waist decreased; autonomous urination function did not recover.

Follow-up thoracic spine MRI demonstrated partial reduction of intramedullary inflammatory enhancement; these imaging changes occurred during combined immunosuppressive therapy and cannot be solely attributed to MMF. The presence of spinal cord cystic or myelomalacic changes suggests potentially irreversible parenchymal injury, which may underlie the patient’s incomplete neurological recovery.

#### Tumor outcome

After restarting pemetrexed combined with carboplatin chemotherapy, the patient completed two cycles of chemotherapy. Recent evaluation showed SD, with no enlargement of left pleural lesions, no significant increase in pleural effusion, and no new lesions.

### Drug safety and long-term prognosis

During treatment, the patient had no MMF-related gastrointestinal discomfort, myelosuppression, or liver and kidney function damage, and steroid adverse reactions were mild. Currently, he is still receiving maintenance treatment with MMF 0.5 g PO BID + prednisone 20 mg PO QD. It is planned to gradually reduce the steroid dose according to the recovery of neurological function and maintain MMF for a long time to prevent recurrence of inflammation; at the same time, chemotherapy will be continued to control the tumor, thoracic spinal MR and tumor imaging will be repeated regularly, and neurological function and tumor progression will be followed up long-term.

## Discussion

This case illustrates a diagnostically complex presentation of probable immune checkpoint inhibitor-associated TM. In accordance with the 2023 SITC consensus terminology for irAEs, we define this presentation as steroid-unresponsive (10). Several overlapping potential contributing factors were identified, including serum anti-Ri paraneoplastic antibody positivity and Epstein–Barr virus sequences detected within the cerebrospinal fluid. Alternative etiologies, including leptomeningeal malignancy, postprocedural inflammatory myelopathy, EBV-driven primary CNS infection, and paraneoplastic-mediated myelitis, were comprehensively evaluated.

Leptomeningeal malignancy is clinically very unlikely, supported by negative CSF cytology for malignant tumor cells and the absence of leptomeningeal enhancement on serial gadolinium-enhanced brain-spine MRI. Postprocedural inflammatory myelopathy is also improbable, given that intrathecal pain-relieving intervention was performed approximately 1 month before symptom onset, inconsistent with the typical time course of procedure-related spinal injury. Although EBV sequences were detected in CSF-NGS, there was no clinical or laboratory evidence supporting overt active central nervous system infection, indicating that EBV most likely acts as a potential immune-activating cofactor rather than the primary pathogenic driver. This complex case was reviewed by a multidisciplinary team comprising oncologists, neurologists, and radiologists. The multidisciplinary consultation favored ICI-driven immune dysfunction as the predominant pathogenic mechanism, while mixed multifactorial contributions could not be fully ruled out.

This case shares several features with previously reported ICI-associated TM, while also presenting unique clinical facets. Firstly, the timing of onset is consistent with ICI-associated irAEs. The patient developed the disease after receiving four cycles of nivolumab combined with ipilimumab, within the period of highest incidence of irAEs (median, 6 weeks). Although he received a cycle of chemotherapy during this period, severe spinal cord involvement occurred only 1 week after chemotherapy, which is highly consistent with the typical timing of ICI-associated irAEs, making chemotherapy-induced neurotoxicity less likely (11). Literature reports show that the onset of ICI-associated TM mostly occurs within 1–74 weeks after treatment, with 80% occurring within the first 4 months, consistent with the timing of onset in this case. Secondly, the clinical manifestations are typical, with abnormal sensory level, limb weakness, and urinary retention as the principal symptoms. The neurological examination is highly consistent with the spinal cord lesion segments. Spinal MRI demonstrates longitudinally extensive inflammatory lesions, and cerebrospinal fluid shows elevated protein and positive oligoclonal bands, findings consistent with the typical auxiliary examination features of ICI-associated Longitudinally Extensive Transverse Myelitis (LETM) (3). Among the 10 cases of ICI-associated LETM reported by Picca et al., eight cases presented with limb weakness, sensory disturbance, and sphincter dysfunction, and seven cases had elevated cerebrospinal fluid protein. These findings are consistent with the manifestations of this case (3, 6, 12–14).

However, a unique feature of this case is the positivity for both anti-Ri antibody IgG and EBV. Paraneoplastic syndromes associated with anti-Ri antibodies are mostly related to small-cell lung cancer, typically presenting with cerebellar degeneration, brainstem encephalitis, and similar manifestations. Cases with predominant syringomyelia involvement are relatively rare. Isolated spinal cord involvement due to EBV infection is also extremely rare; when it occurs, lesions are usually short-segment rather than long-segment spinal cord lesions. In addition, paraneoplastic syndrome may show either spinal cord enhancement or no enhancement on imaging, making it difficult to distinguish from other etiologies based on radiological findings alone. Immune checkpoint inhibitors may induce anti-Ri antibody-mediated spinal cord injury by activating underlying autoimmune responses. According to multidisciplinary consensus, both EBV infection and paraneoplastic syndrome may serve as additional triggers that induce or exacerbate immune-mediated responses. Nevertheless, the predominant pathogenic mechanism in this case remains immune-related adverse events associated with immune checkpoint inhibitors. Long-term follow-up is warranted to assess the impact of these findings on the patient’s prognosis.

Currently, no established standard exists for treating steroid-unresponsive ICI-associated TM. High-dose glucocorticoid pulse therapy is recommended as first-line treatment, whereas the choice of second-line treatment should be guided by case reports and expert consensus. Existing second-line treatment options reported in the literature mainly include IVIg, plasmapheresis, cyclophosphamide, rituximab, and other immunosuppressive therapies. Among the four cases of ICI-associated TM reported by Chatterton et al., three cases received IVIg or plasmapheresis after steroid treatment failed, and two cases had partial recovery of neurological function (3). Charabi et al. reported one case of pemetrexed-related LETM, whose symptoms were relieved after plasmapheresis combined with steroid treatment but relapsed after drug withdrawal (15). Davis et al. reported a case of steroid-refractory immune checkpoint inhibitor-associated TM. The patient achieved complete neurological recovery after high-dose cyclophosphamide treatment, with no recurrence during 6 years of follow-up (6). Picca et al. demonstrated that marked elevation of cerebrospinal fluid IL-6 is closely associated with severe steroid-refractory ICI-related myelitis, and targeted therapy with anti-IL-6 receptor agents plus JAK inhibitors can effectively improve neurological function and radiological abnormalities (9). Otto et al. reported one case of ICI-associated LETM with elevated cerebrospinal fluid CXCL13, in which the patient’s condition remained stable after rituximab treatment (7). Overall, for patients with steroid-refractory ICI-associated TM, those who received timely additional immunosuppressive therapy had a favorable overall prognosis (6, 7, 9). In contrast, cases reported in the literature that were steroid-refractory and unresponsive to glucocorticoids or intravenous immunoglobulin and that did not receive targeted immunosuppressive agents regardless of biomarker status generally exhibited a poor prognosis (3, 13, 15).

The choice of immunosuppressive therapy for this case was mainly based on the following considerations. First, the patient had a steroid-unresponsive immune-related adverse event (irAE), with only minimal meaningful neurological benefit from first-line high-dose glucocorticoid pulse therapy combined with IVIg. According to previous literature, immunosuppressive agents should be initiated as early as possible in patients with severe ICI-associated TM or those with poor response to first-line therapy. Second, the etiology of this case was complex and required a comprehensive approach targeting multiple immune pathways. Rituximab, as reported in the literature, mainly targets B cells and has limited efficacy against T-cell-mediated inflammation. The patient presented with concurrent ICI-related immune injury, paraneoplastic syndrome (positive anti-Ri antibody), and positive cerebrospinal fluid oligoclonal bands, which could not be fully addressed by single targeted inhibitors. Third, MMF offers superior safety and clinical applicability for long-term maintenance therapy. Although cyclophosphamide, used in severe cases, acts rapidly, it carries a high risk of myelosuppression and gonadal toxicity. Since the patient required continuous antitumor chemotherapy, agents with significant myelosuppression and severe side effects were avoided. MMF inhibits inosine monophosphate dehydrogenase and blocks the purine synthesis pathway, thereby specifically suppressing the proliferation and activation of T and B lymphocytes. It is also recommended as a second-line immunosuppressive agent for irAEs in the National Comprehensive Cancer Network (NCCN) Guidelines for Immune-Related Adverse Events. In addition, oral MMF is convenient to administer, with relatively mild adverse reactions such as myelosuppression. The dosage can be adjusted through regular monitoring of blood counts, liver and renal function, making it more suitable for long-term maintenance. Real-world experience with MMF for ICI-related myelitis remains limited, and one prior case used MMF for steroid-sparing maintenance after disease relapse (16).

In the present case, reduction of spinal inflammatory signals and partial neurological improvement were observed under multimodal immunosuppression consisting of reescalated corticosteroids and MMF, without obvious MMF-related adverse reactions. Given that clinical improvement cannot be disentangled from the concurrent adjustment of corticosteroid dose, we cannot draw definitive conclusions regarding standalone MMF efficacy. Nevertheless, considering the minimal clinical benefit achieved with preceding high-dose glucocorticoid pulse plus IVIg, the greater clinical improvement observed after MMF initiation indirectly suggests that MMF may have contributed to the therapeutic response in this individual case. While our single-case observation indirectly suggests the potential utility of MMF in steroid-unresponsive ICI-associated TM, it cannot validate MMF as an established therapeutic option for this neuro-irAE. MMF may reasonably be discussed as a steroid-sparing maintenance option; however, the present case does not establish its efficacy as an acute rescue treatment. Drawing on NCCN guidance on immune-related adverse events in other organ systems and limited real-world case experience, MMF may be considered an alternative second-line option in similar complex clinical scenarios; further multicase clinical data accumulation is required to establish its clinical value for ICI-associated TM.

## Conclusion

In summary, we report a diagnostically complex case of probable steroid-unresponsive ICI-associated TM with multiple potential immune-activating cofactors, including serum anti-Ri antibody positivity and EBV sequences detected in CSF. Partial neurological recovery and partial radiological improvement were achieved during multimodal immunotherapy combining reescalated corticosteroids and mycophenolate mofetil. The independent therapeutic contribution of MMF cannot be reliably disentangled from the effect of concurrent corticosteroid adjustment; therefore, definitive efficacy of MMF for acute rescue in steroid-unresponsive ICI-associated TM cannot be established by this single-case observation. Further cumulative clinical data are needed to define the role of mycophenolate mofetil in this rare neuro-irAE.

## Study limitations

This is a single-case report with a relatively short follow-up duration. The risk of recurrence of ICI-associated TM may persist beyond 1-year posttreatment. Accordingly, the long-term efficacy and safety profile of MMF in this setting remain to be confirmed. Given the coexistence of serum anti-Ri-IgG positivity and EBV sequences detected in CSF, complete exclusion of contributions from multifactorial immune activation is not feasible, and only a probable diagnosis of ICI-associated TM can be established rather than definitive causal attribution. Published real-world experience regarding MMF for ICI-associated TM remains scarce. Direct comparative evidence between MMF and alternative second-line immunosuppressive agents, including cyclophosphamide and rituximab, is lacking. Further multicenter case series are required to better define the optimal therapeutic role of MMF for this rare neuro-irAE.

## Data Availability

The original contributions presented in the study are included in the article/Supplementary Material. Further inquiries can be directed to the corresponding authors.
